# The Association between Palmer Drought Severity Index Data and Tuberculosis-like Lesions Occurrence in Mediterranean Hunted Wild Boars

**DOI:** 10.3390/ani11072060

**Published:** 2021-07-10

**Authors:** Ana Carolina Abrantes, João Serejo, Madalena Vieira-Pinto

**Affiliations:** 1 CECAV-Animal and Veterinary Research Centre, Trás-os-Montes e Alto Douro University, Quinta de Prado, 5001-801 Vila Real, Portugal; mmvpinto@utad.pt; 2 Câmara Municipal de Idanha-a-Nova, 6060-163 Idanha-a-Nova, Portugal; al51613@utad.eu; 3 Department of Veterinary Science, Trás-os-Montes e Alto Douro University, Quinta de Prados, 5001-801 Vila Real, Portugal

**Keywords:** MTC, drought, Mediterranean climate, mycobacteria, Portugal, tuberculosis

## Abstract

**Simple Summary:**

Climate is one of the most influential factors in the dynamics of tuberculosis in the Mediterranean Iberic wildlife population. In this study, we aim to address how drought as a risk factor influences the occurrence of Tuberculosis-like lesions (TBL) in wild boar inspected in the field. With this focus, our study contributes to pointing out the importance of periods of drought in the increased TBL occurrence in wild boars inspected in the field in the subsequent season. The results of our study allow hunting managers to be advised, in advance, on whether they should adopt extra protective measures when they are aware of the presence of periods of drought. This climate trait can become an alert sign for increased TBL occurrence in the following season, allowing for the implementation of a feasible, timely, and effective measures to control TB in the wild boar population.

**Abstract:**

In the Iberian Peninsula, the prevalence of tuberculosis differs for each region and for different wild disease hosts and the region affected by a Mediterranean climate will be the most affected. The Mediterranean Iberic regions have a favourable ecosystem for the development of Mycobacterium tuberculosis complex agents, where habitat, population dynamics, and climate (especially drought) are important factors affecting the high prevalence of tuberculosis in the wild boar population. Our objective was to study the association between the Palmer Drought Severity Index (PDSI) and the occurrence of tuberculosis-like lesions (TBL) in wild boar during nine hunting seasons (2008/09 to 2016/17) in Idanha-a-Nova County. To this end, statistical analysis revealed a significant association (*p* < 0.05) between the occurrence of TBL in wild boar in Idanha-a-Nova County and the analysed risk factor—previous season with periods of drought—which indicated that, when one season experiences some periods of drought, the probability of TBL occurrence in wild boars was 1.2 (OR = 1.2) times higher in the next hunting season than when there were no periods of drought. Therefore, our study contributes to the discovery of a positive effect of periods of drought on the transmission of tuberculosis in Iberian wildlife.

## 1. Introduction

Animal tuberculosis (TB) is a chronic zoonotic disease caused by Mycobacteria species which belongs to the Mycobacterium tuberculosis complex (MTC), such as *Mycobacterium bovis* and/or *Mycobacterium caprae,* as well as others that are etiologically linked to human infection [1]. TB has spread worldwide and is considered an emerging disease in wildlife in several areas, with wild reservoirs being one of the potential foci of infection for domestic species and humans [2].

Most of the European-published studies about TB in wildlife have focused on the Iberian Peninsula. In this Peninsula there are different prevalences of TB for each region, where the Mediterranean climate region is the most affected. The Mediterranean Iberic regions have a favourable ecosystem for MTC mycobacteria development where habitat, population dynamics, and climate are important factors in the high prevalence of TB [3,4,5].

In particular, in Portugal in 2018, data have shown a low seroprevalence in wild boar. Santos et al. described a 2.4% prevalence in 678 wild boars in two Portuguese geographic clusters; however, when only the second cluster was analysed, the seroprevalence increased to 12.3% [6]. This cluster encompasses the central east region of Portugal, which includes Idanha-a-Nova County, which is the place of the present study. In this region, there exists a well-organised multi-host system in which domestic species (e.g., bovines, goats, and sheep) and game species participate and where wild boar (*Sus scrofa*) and red deer (*Cervus elaphus*) play special roles [7,8]. One of the hypotheses raised in several studies is the fact that the existence of permanently TB infected game species are the cause of the failure in the eradication of TB in cattle in this area where there are several confirmed infected cattle hotspots.

In 2011, previous studies have related a consistently higher TB infection rate in wild boar (16–35%) than in red deer (7–10%), with laboratorial confirmation, in Idanha-a-Nova County [8], to which several main factors have been involved. A multifactorial system contributes to this TB prevalence and dynamic in wild boars, such as age, sex, interface with other species (e.g., red deer or cattle), presence of co-infectious diseases, and artificial supplementation by hunting managers [9,10].

The disease chronicity and related morbidity of TB have been associated with a constant and regular *M. bovis* circulation and this a fact which makes the interpretation of the patterns of the disease more difficult [11]. This, together with a wild boar population that is spread throughout the Iberian Peninsula and that is extremely susceptibility to TB, turns them into a true reservoir and an excellent indicator of TB presence in Mediterranean wildlife [12,13]. The proportion of tuberculosis-like lesions (TBL) observed during sanitary inspection of large game during the hunting season is one of the best methods to (indirectly) evaluate the prevalence of TB in wild boar [13]. TBL data are indirect extrapolation data, always lacking laboratory confirmation such as PCR and bacteriological culture to confirm TB infection [14]. In the case of TB high-risk areas, such as Idanha-a-Nova County, the TBL occurrence is a useful epidemiological tool even without laboratory confirmation.

The wild boar has interesting behavioural characteristics since it is nocturnal and possesses great ease of adaptation to the environment that surrounds it, permitting it a great capacity to move and cross large barriers (e.g., rivers, motorways, and fences). This capacity has been associated to its interface and indirect or direct contact with other animals, livestock, or wildlife, promoting the transmission of mycobacteria, especially at risk points such as waterholes and artificial feeders [15,16]. The international literature has reported a mixture of direct oral-nasal transmission and indirect transmission induced by feeding and watering locations where MTC mycobacteria can survive for long periods [5,17,18].

Times of food scarcity in wild boar habitats, originating from anthropogenic alterations or climate changes, may promote this food demand behaviour elsewhere resulting in possible aggregation at risk points [11].

Our objective was to study the association between Palmer Drought Severity Index (PDSI) data and the occurrence of tuberculosis-like lesions (TBL) in the wild boar population during nine hunting seasons (2008/09 to 2016/17) in Idanha-a-Nova County. Consequently, we extrapolate the effect that occurs in the dry season on the TBL dynamic in hunted wild boar and, in allowing hunting managers to handle this issue in the best banner, thus increase the preventive measures in such seasons.

## 2. Materials and Methods

### 2.1. Area of Study

**Figure 1:**
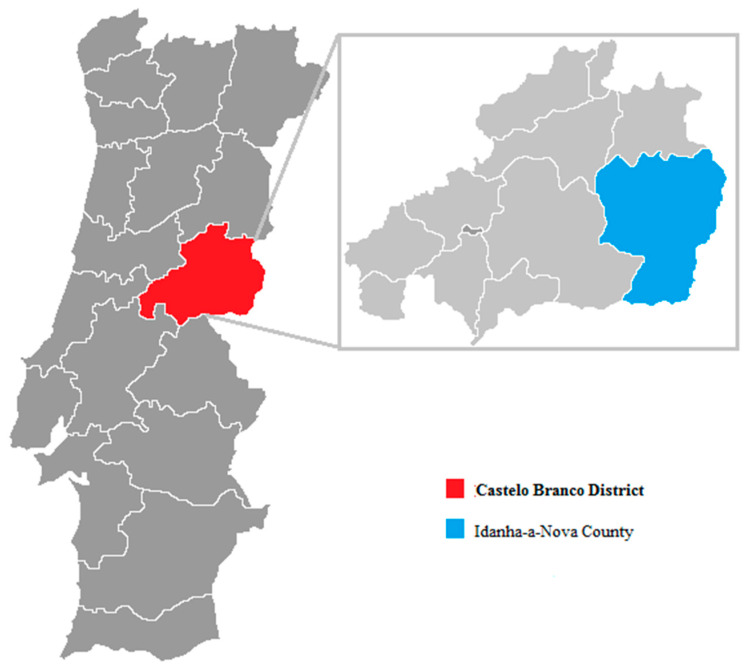
Idanha-a-Nova County in a map of Portugal.

The study was developed in Idanha-a-Nova County (Figure 1), which is located in central east Portugal (lat 39°55′ N; long 7°14′ W; 1416.0 km^2^) in Castelo Branco District (Portugal). To the east, Idanha-a-Nova County is delimited by the river Erges and, to the south, by the river Tagus; these two rivers form the border with Spain (Extremadura region) [19].

Geographically, Idanha-a-Nova is a flat county on which many forms of relief are imposed; a real transition of landscape occurs from the northern mountains to the southern plateau of Portugal. The climate is characterised by a temperate Mediterranean climate (hot dry summers and cold rainy winters), but with thermal amplitudes strongly marked by the seasonality [20].

The geographic and climatic characteristics offers advantages for both agricultural and animal production activities due to the presence of sun and moderate rain as well as for outdoor sports, such as hunting activities. In fact, this county is known as one of the best places to hunt in Portugal and large game hunting is the most significant here. Overall, 86% of the county, which corresponds to 120,000 ha, make up the hunting area [21].

### 2.2. Data Collection

Idanha-a-Nova is in the epidemiologic high-risk area for bovine tuberculosis in large game where, in 2011, the Portuguese Veterinarian Authority published an internal law (Edital No. 1) describing mandatory rules that must be observed during each driving hunt organised in this Epidemiologic Risk Area [22]. This is a high-risk area with high values of TB infection in game species and related TB infected cattle hotspots [9,10].

In each drive hunting action, the wild boars hunted are collected and gathered in an evisceration area where a veterinarian carries out sanitary evaluation (referred to as an initial examination) of wild boars on the spot, as legislated in Reg. 853/2004. Briefly, this systematic initial examination in loco includes incision of the lymph nodes, such as mandibular, prescapular, pulmonary, and mesenteric and the inspection of the animal’s viscera [23]. A TBL is defined as all caseous or caseocalcareous tubercules with different sizes presenting in the lymph nodes and viscera [24,25].

In this study, data of wild boar rejection for consumption due to TBL during nine hunting seasons (2008/09 to 2016/17) were analysed by the same veterinarian for all seasons with the same modus operandi, where an animal was considered positive when at least one TBL was found and the carcass was rejected in full. In this study, we sampled 3923 wild boars from a total of 524 drives during the nine different hunting seasons.

The climate data studied were organised in a database according to public data of the Instituto Português do Mar e da Atmosfera, I. P. (IPMA, I. P.), a Portuguese state organisation with responsibilities at the level of the national territory in the domains of the sea and atmosphere. The main climate index is the PDSI (Palmer Drought Severity Index), which is based on the concept of water balance and based on taking into account data on the amount of precipitation, air temperature, and available water capacity in the soil. This index allows for the detection of periods of drought and classifies them in terms of intensity (weak, moderate, severe, and extreme) [26].

In this study, we analysed the influence of a previous season that had periods of drought as a possible risk factor for increased wild boar TBL occurrence in the following hunting season.

### 2.3. Statistical Analysis

This study was an uncontrolled cross-sectional study that was undertaken with data collected in field during nine hunting seasons. To detect “previous season with periods of drought” as a possible risk factor for TBL occurrence in wild boar, we established a two-by-two table.

For the analysis, the following were used: odds ratio (which measures the association between an exposure and a result) and Fisher’s exact Test *p*-values. A probability value (*p*-value) < 0.05 was considered statistically significant.

Both values were calculated using the EpiTool version 0.5-6 statistical analysis software.

## 3. Results

**Figure 2:**
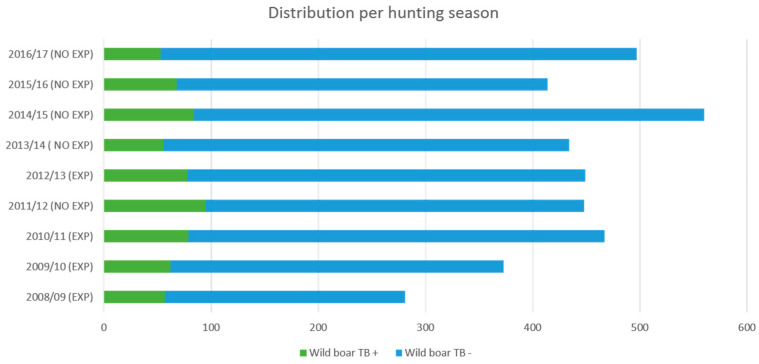
Distribution per hunting season (exposed/non-exposed) of wild boar that were TBL positive (green) and TBL negative (blue).

Of the nine hunting seasons analysed, four were exposed to the risk “previous season with periods of drought,” while the other five seasons were not exposed. A total of 3923 wild boar in the nine seasons were analysed: 1570 wild boars in the four hunting seasons were exposed (2008/09, 2009/10, 2010/11, 2012/13; 40.0%) and 2353 wild boars in the five hunting seasons were non-exposed (2011/12, 2013/14, 2014/15, 2015/16, 2016/17; 60.0%; see Figure 2).

**Figure 3:**
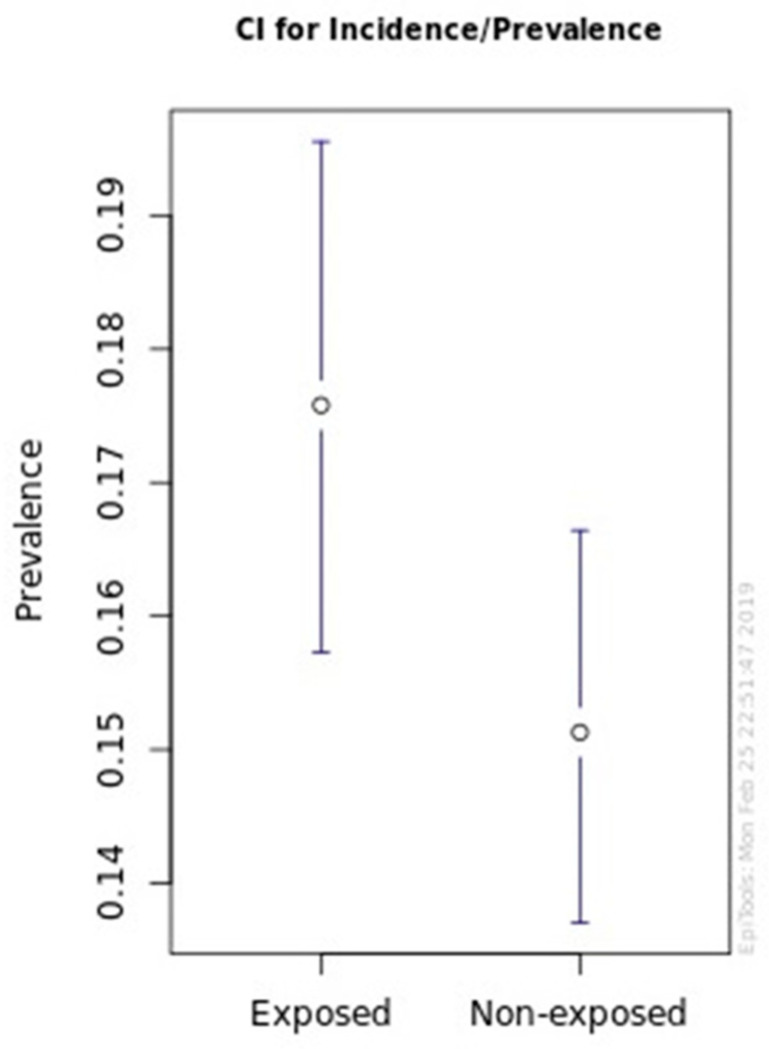
Graphics with wild boar TBL occurrence in exposed and non-exposed hunting seasons.

In the four hunting seasons exposed to the risk, 276 of 1570 wild boars were detected with TBL during the sanitary inspection (17.6%) and the remaining 1294 wild boars were without TBL (82.4%). On the other hand, in the five hunting seasons without exposure to a previous dry season, we found 356 of 2353 wild boars with TBL (15.1%) and 1997 wild boars without TBL (84.9%). The graph with the estimated incidence of TBL (Figure 3) demonstrates the previously explained distribution.

The risk factor “previous hunting season with periods of drought” revealed a statistically significant association (*p* < 0.05) with the incidence of TBL in wild boars in Idanha-a-Nova County during the nine hunting seasons considered in the study. We found that when one hunting season had some periods of drought, the probability of TBL incidence in wild boars was 1.2 (OR = 1.2) times higher in the next hunting season compared to when there were no periods of drought.

## 4. Discussion

It is worth noting that all TBL detected in the 3923 wild boars analysed may not have been real TB lesions caused by MTC mycobacteria, as these lesions were macroscopic lesions observed in field and we had no laboratory confirmation of MTC agent presence [2,4]. The gold standard of TB confirmation is bacteriological culture [2] since macroscopic lesions may be the result of other agents, such as Actinomyces, *Rodococcus equi*, or other mycobacteria (e.g., *Mycobacterium avium*) [4]. However, in this area of study and in several cases, the isolation of *M. bovis* in game species was confirmed [7,9].

However, it is well-known that the post-mortem inspection of wild ungulates in the field after drives hunting is one of the most widely used, affordable, and inexpensive methods to estimate the occurrence of TB in the wild population, including game species such as wild boar [4].

One of most-discussed risk factors involved in TB transmission in wildlife species or between cohabitant species, such as cattle and game, is the climate. The influence of the Mediterranean climate has been described as one of the factors which modulates the epidemiology of TB in wildlife population in Iberian Peninsula [27], including its presence in the wild boar population in the southeast of central Portugal such as the Idanha-a-Nova County [5,10].

One of the peculiarities of climatic influence in TB transmission and dynamic in wildlife is that the positive/negative effect on the hosts is long-term due to the chronicity of TB infection [28]. The climate effect results were not immediately on same time scale with respect to the climate alterations occurred; TB dynamics only changed in the next seasons. Our study was based on this principle since we considered that the TBL occurrence in wild boars in Idanha-a-Nova was dependent on the drought effect not during the hunting season with respect to data collection, but on the prior hunting season.

Our results are in accordance with those of Vicente et al. [4,11], who described a statistically significant increase in TB-infected wild boar with a decrease in rain in the previous season and described an increase in TBL in years with periods of drought.

The climate influences some environmental characteristics which, in turn, influences the TB dynamics in the wild boar population. The persistence of *M. bovis* in the environment, amount of food available, animal body condition, and the anthropogenic actions (e.g., the management of artificial food supplementation to game differs seasonally) may be factors that influence the persistence of the infectious agent in the wild boar population.

Mycobacteria such as *M. bovis* can survive in the environment (water, soil, and hay) over a period of months [11,12,13,14,15,16,17]. Some studies have shown that the survival of *M. bovis* in the environment depends on low temperature, low sun exposure, dark and humid areas, and soil organic proprieties [29]. Increases in the capacity of *M. bovis* survival in the environment and of the infection potential are observed especially in humid, less sunny, and cold seasons, which could be questioned due to the contradiction with our results.

This occurred due to the amount of food available in the field and wild boar’s body condition being related; the periods with drought decrease the food available and, consequently, a loss of body condition in wild boars is observed [30]. Such food restrictions are more limitative for the wild boar than other wildlife ungulates (e.g., red deer) due to the fact that the wild boar is not a territorial species [11]. In the dry season, the wild boar’s body condition decreases, and they become more immunodepressed and weak [30]. They are more vulnerable to infections and, consequently, to TB infection. This TB infection can result from risk point aggregation or contact with TB-infected cohabitant animals or predators [11,30]. Aggregation at risk points, such as waterholes, increases in dry periods; that is, when nutrition sources are scarce and wild boars look for other water and food sources in other territories. The contact frequency in these scarce natural points increases in warm and dry seasons such that the risk of direct or indirect transmission of TB between ungulates is higher [31].

Anthropogenic actions fluctuate with the season [28]. The wild boar possesses good response to the artificial food supplementation. With this knowledge, farmers and hunters in the warm and dry season usually offer artificial food supplementation to cattle and game, with the objective of minimising the decrease in the animal’s body condition. As a consequence of this, an overabundance of wildlife can be observed in Mediterranean area [32].

Artificial food supplementation and the locale where farmers carry it out are crucial for increasing (intra-species and inter-species) TB transmission due to animal aggregation at these artificial water and feed points, which magnifies the probability of transmission [27].

In short, in the Mediterranean habitat (inclusive of Idanha-a-Nova County and Portugal in general) for wild boar, the climate—more specifically drought—can be considered a risk factor for TB infection. A natural phenomenon of summer droughts (periods included in the normal hunting season) often occurs in Mediterranean climate regions when food and water become scarce. Consequently, taking into consideration wild boar overabundance and increased artificial feeding and watering provided by hunters [28,29,30,31,32,33], the conjunction of these three factors (drought, artificial supplementation, and overpopulation) result in wild boar aggregation at risk points and potent TB infection in Portugal, especially in the Idanha-a-Nova County. Our study contributes to the body of knowledge by demonstrating that periods of drought increased the occurrence of TBL in wild boars inspected in the field in Idanha-a-Nova County during nine hunting seasons (2008/09 to 2016/17), thus indicating a positive effect of periods of drought on the transmission of TB.

## 5. Conclusions

Our study contributed to the body of knowledge by pointing out the importance of periods of drought in the increased TBL occurrence in wild boars inspected in field during the subsequent season. This result allows hunting managers to be advised in a timely manner of when to adopt extra protective measures; that is, every time they are aware of the presence of periods of drought, they will be able to adopt measures. This climate trait can become an alert sign for the increase in TBL occurrence in the next season, allowing the implementation of feasible, effective, and timely measures to control TB in the wild boar population.

In traditional hunting areas and areas that are simultaneously TB high-risk hotspots, some preventive game management actions are common during all seasons. With this study, we suggest an improvement of the preventive measures with the exclusion of food supplementation in high-risk seasons or/and to adopt extra TB preventive measures in these aggregation points.
